# Perioperative Fluid Management and Renal Protection in Cytoreductive Surgery with Hyperthermic Intraperitoneal Chemotherapy (CRS/HIPEC): A Scoping Review

**DOI:** 10.3390/jcm15187307

**Published:** 2026-09-20

**Authors:** Gilda Pasta, Camilla L’Acqua, Nicoletta Filetici, Giulia Bernardi, Arturo Cuomo, Edoardo De Robertis, Tiziana Bove, Rachele Simonte, Luciano Frassanito

**Affiliations:** 1 Department of Anesthesiology, Pain Therapy and Intensive Care, INT IRCCS Fondazione G. Pascale, 80131 Naples, Italy; g.pasta@istitutotumori.na.it (G.P.); a.cuomo@istitutotumori.na.it (A.C.); 2 Department of Anesthesia and Intensive Care, Fondazione IRCCS Istituto Nazionale dei Tumori, 20133 Milan, Italy; camilla.lacqua@istitutotumori.mi.it; 3 Department of Science of Emergency, Anesthesiology and Intensive Care, IRCCS Policlinico A. Gemelli Foundation, 00168 Rome, Italy; nicoletta.filetici@policlinicogemelli.it (N.F.); giulia.bernardi@policlinicogemelli.it (G.B.); tiziana.bove@policlinicogemelli.it (T.B.); 4 Department of Medicine and Surgery, University of Perugia, 06122 Perugia, Italy; edoardo.derobertis@unipg.it (E.D.R.); rachele.simonte@gmail.com (R.S.); 5 Department of Basic Biotechnological Sciences, Intensive Care Peri-Operative Clinics, Università Cattolica del Sacro Cuore, 00168 Rome, Italy

**Keywords:** cytoreductive surgery, hyperthermic intraperitoneal chemotherapy, perioperative fluid therapy, goal-directed fluid therapy, acute kidney injury, nephroprotection, haemodynamic monitoring, peritoneal carcinomatosis

## Abstract

**Background/Objectives:** Cytoreductive surgery combined with hyperthermic intraperitoneal chemotherapy (CRS/HIPEC) is a complex, high-risk procedure associated with profound haemodynamic and metabolic perturbations. Perioperative fluid management and the risk of acute kidney injury (AKI) represent two of the most clinically significant and mechanistically interrelated anaesthetic challenges in this setting. This scoping review aimed to synthesise the current evidence on perioperative fluid therapy strategies and renal protection in CRS/HIPEC, examining the pathophysiological rationale, comparative outcomes of different fluid therapy strategies, haemodynamic monitoring modalities, AKI incidence and risk factors, and pharmacological nephroprotective strategies. **Methods:** The review followed PRISMA guidelines and was registered in the Open Science Framework (osf.io/864pr). Studies specifically addressing fluid management, haemodynamic monitoring, AKI, and nephroprotection in adult patients undergoing CRS/HIPEC were included. **Results:** Thirty-one studies met inclusion criteria across two thematic domains: 13 addressing fluid therapy (two RCTs, one systematic review, and 10 observational studies) and 18 addressing AKI and renal protection (two RCTs, one systematic review, and 15 observational studies). Liberal intraoperative fluid administration is independently associated with increased morbidity and prolonged hospital stay. Restrictive and goal-directed strategies appear safe and are associated with improved outcomes. AKI incidence ranges from 7.9% to 45.5% depending on chemotherapy regimen, with platin- and taxane-based HIPEC conferring the highest risk. Sodium thiosulfate, amifostine, and cilastatin demonstrate nephroprotective effects in retrospective cohorts; dexmedetomidine shows subclinical renal biomarker benefits in the only RCT identified. **Conclusions:** The available evidence supports restrictive and goal-directed fluid strategies guided by dynamic haemodynamic monitoring, alongside individualised, cisplatin-dose-adapted nephroprotective protocols. Both fluid overload and renal hypoperfusion represent modifiable risk factors for AKI in CRS/HIPEC. Prospective randomised trials integrating fluid management and renal outcome endpoints are required.

## 1. Introduction

Cytoreductive surgery (CRS) combined with hyperthermic intraperitoneal chemotherapy (HIPEC) has emerged as the standard of care for selected patients with peritoneal surface malignancies, including colorectal, ovarian, and gastric cancer peritoneal metastases, pseudomyxoma peritonei, and peritoneal mesothelioma [1,2,3,4,5,6,7,8,9].

By combining maximal surgical tumour debulking with the direct delivery of heated chemotherapy to the peritoneal cavity, the procedure exploits both the synergistic cytotoxicity of thermal potentiation and the pharmacokinetic advantage of regional drug concentration. In appropriately selected patients, CRS/HIPEC has been associated with significantly prolonged overall survival compared with systemic chemotherapy alone [1,2].

However, CRS/HIPEC ranks among the most physiologically demanding procedures in oncological surgery. Typical operative times range from 6 to 18 h; peritoneal stripping and multi-visceral resections impose haemorrhagic, inflammatory, and coagulative stress; and the hyperthermic perfusion phase induces a hyperdynamic, vasodilatory circulatory state with increased metabolic demands [10,11,12,13,14,15]. The complexity and duration of these insults subject patients to a sequence of haemodynamic, thermoregulatory, electrolytic, and nephrotoxic perturbations that unfold across distinct procedural phases, each with its own anaesthetic management implications [11].

Among the many perioperative management challenges, two stand out for their clinical relevance and mechanistic interconnection: perioperative fluid therapy and acute kidney injury (AKI). Fluid overload has been consistently associated with increased morbidity in major abdominal surgery, yet the profound fluid losses inherent to CRS/HIPEC have historically driven liberal administration practices [1]. Concurrently, AKI is one of the most frequent and prognostically significant postoperative complications of this procedure, driven by a convergence of haemodynamic, thermal, and direct nephrotoxic effect, particularly in the context of cisplatin-based HIPEC regimens [15].

Despite their clinical importance and pathophysiological interdependence, these two domains have predominantly been addressed separately in the literature. The present scoping review aims to provide an integrated, academically balanced synthesis of the evidence on both topics, examining: (1) the pathophysiological basis of fluid and haemodynamic perturbations across the phases of CRS/HIPEC; (2) the comparative evidence for liberal, restrictive, and goal-directed fluid strategies; (3) the role of haemodynamic monitoring modalities; (4) the incidence, risk factors, and definitions of AKI in this setting; and (5) pharmacological and non-pharmacological nephroprotective strategies. The review concludes with a unified discussion identifying shared clinical priorities, evidence gaps, and directions for future research.

A scoping review methodology was specifically chosen over a systematic review due to the marked clinical heterogeneity of the CRS/HIPEC literature—spanning diverse primary tumour histologies, heterogeneous cytoreductive extents, varied intraperitoneal chemotherapy regimens, and predominantly observational study designs with limited RCTs. The primary objective was to map the broad conceptual nexus between perioperative fluid dynamics and nephroprotection, rather than computing a single aggregated meta-analytic effect size.

## 2. Materials and Methods

This review was conducted in accordance with the Joanna Briggs Institute (JBI) methodological guidance for scoping reviews and is reported according to the Preferred Reporting Items for Systematic Reviews and Meta-Analyses extension for Scoping Reviews (PRISMA).

The project began as a narrative synthesis of perioperative management in CRS/HIPEC. During the screening phase it became apparent that the volume and heterogeneity of the retrieved literature warranted a formal, reproducible mapping exercise, and the work was converted to a scoping review conducted according to JBI guidance and reported per PRISMA-ScR. The protocol was formalised and deposited on the Open Science Framework at that point (project osf.io/864pr; protocol https://doi.org/10.17605/OSF.IO/V9CEA), which was after the initial searches had been run. We state this sequence explicitly rather than presenting the registration as prospective. The deposited document sets out the review question, eligibility criteria, information sources, search strings and data-charting fields as they were applied; the searches were not re-run, extended or narrowed after deposition, and the review is reported in full against the PRISMA-ScR checklist (Supplementary Table S1).

**Figure 1:**
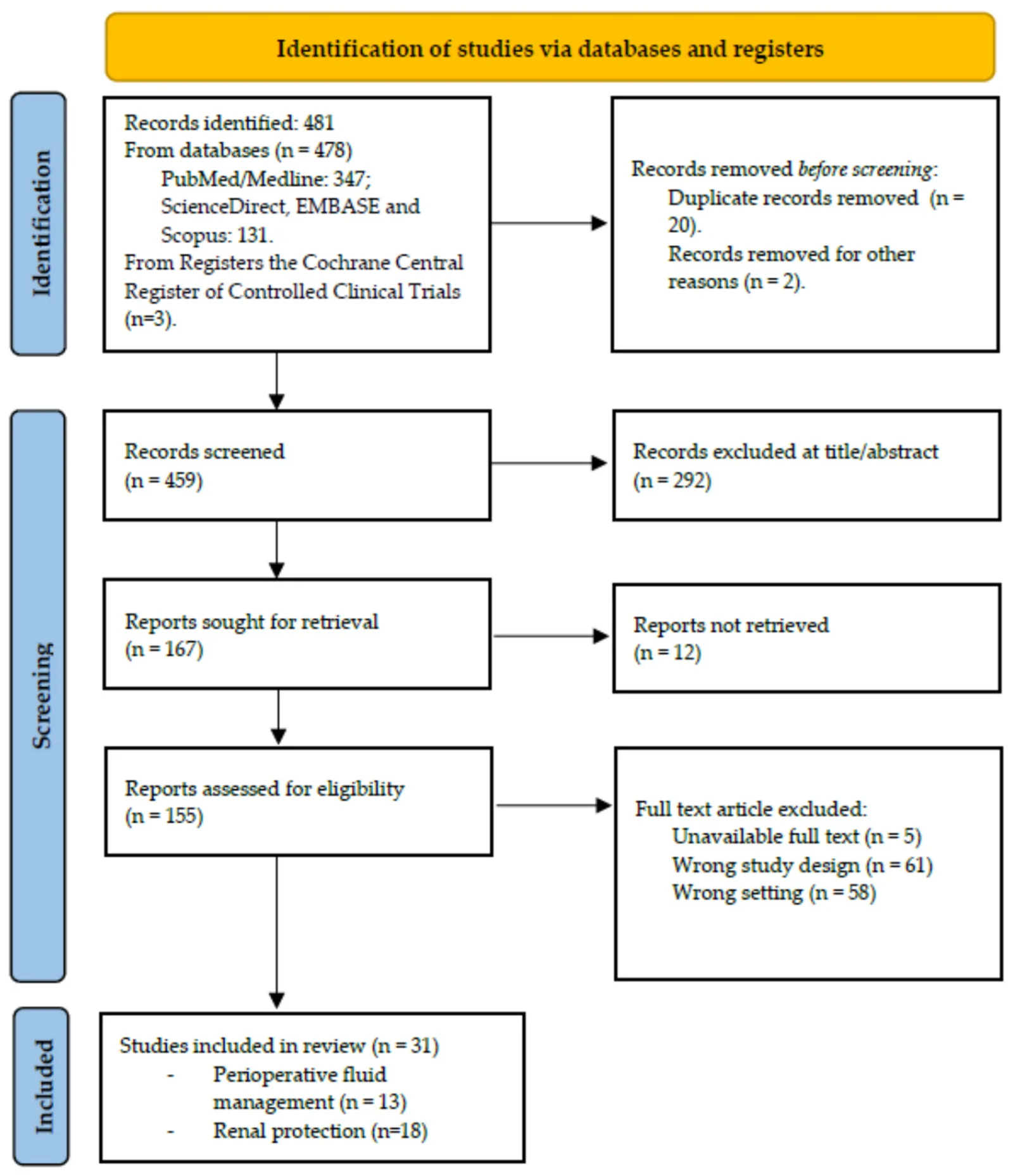
PRISMA-ScR flow diagram of the search and selection process. Sources of evidence cascade from 481 identified records through de-duplication, title-and-abstract screening, full-text retrieval and final inclusion.

The study selection process is summarised in the PRISMA-ScR flow diagram (Figure 1).

### 2.1. Search Strategy

The search strategy was designed to identify all relevant studies regarding perioperative management in HIPEC procedures. Electronic searches were performed in PubMed, the Cochrane Central Register of Controlled Clinical Trials, ScienceDirect, EMBASE, and Scopus using the following Medical Subject Headings (MeSH) terms and free-text keywords: “cytoreductive surgery”, “Hyperthermic Intraperitoneal Chemotherapy”, “HIPEC”, “Intraoperative Management”, “anaesthetic management”, “haemodynamic management”, “goal-directed therapy”, “acute kidney injury”, “fluid therapy”, “intraoperative complications”, and “Perioperative and postoperative complications”, with filters applied for human subjects. The complete keyword combinations, Boolean architecture, and database-specific query adaptations are provided in Supplementary Material S1.

These keywords were then modified for each database to maximise the number of results. Searches were restricted to the peer-reviewed literature published in English between 1 January 2014 and 31 December 2025, including early online-ahead-of-print publications indexed prior to formal print issue assignment (accounting for studies catalogued under early 2026 dates). The English-language restriction was applied to ensure standardisation and critical comparability of complex haemodynamic monitoring protocols and validated AKI diagnostic classifications across international reporting standards.

### 2.2. Eligibility Criteria

Studies were selected based on the following PICO criteria:

- Population: Adult patients (>18 years) undergoing CRS combined with HIPEC for peritoneal malignancies (colorectal, ovarian, mesothelioma, pseudomyxoma, and gastric).
- Intervention/Exposure: Perioperative anaesthetic management and intraoperative chemotherapy protocols for CRS-HIPEC (specifically haemodynamic management, fluid balance, pain control, hyperthermia/metabolic management, intraoperative complications, nephrotoxicity, acute kidney injury and/or disease, and renal protection).
- Outcome: The primary outcomes of interest were the incidence of complications related to intraoperative haemodynamic management and the incidence of postoperative AKI, defined according to standard criteria. Secondary outcomes included the need for renal replacement therapy (dialysis), length of stay, and mortality.
- Study Design: Randomised controlled trials (RCTs), prospective observational studies, retrospective cohort studies, systematic reviews, meta-analyses and case series written in English and available in full text.
- Exclusion criteria: Animal studies, studies on experimental or preclinical HIPEC models only, studies addressing only surgical or oncological techniques without describing anaesthetic management, studies in which anaesthetic management is not clearly described, isolated case reports, editorials, letters to the editor, opinion pieces, and abstracts without full text available.

### 2.3. Study Selection and Data Extraction

The authors reviewed all the references obtained through various phases before data extraction. The initial phases entailed abstract screening for the relevance of the articles, after which all irrelevant articles were eliminated. The full articles of remaining papers were then sought after and assessed to determine if they met the eligibility criteria. Those that met our inclusion criteria were then used for the data extraction. Reference lists of identified systematic reviews were hand-searched for additional studies. Titles and abstracts were independently screened by four reviewers (L.F., G.P., C.L.A., and N.F.) under the supervision of R.S.; discrepancies were resolved by consensus.

The extracted data included: study characteristics (author, year, design), procedural details (HIPEC duration, chemotherapy agents), haemodynamic management strategies, and renal outcomes (AKI events, dialysis requirements).

### 2.4. Use of Artificial Intelligence Tools

During the preparation of this manuscript, large language models, specifically Gemini (version 1.5 Pro, Google LLC, Mountain View, CA, USA) and Claude (version 3.5 Sonnet, Anthropic, San Francisco, CA, USA), were utilised exclusively for language editing, grammar harmonisation, stylistic polishing of the drafted text, and structural drafting support for Figures. No AI tools were used for literature screening, data extraction, or synthesis. All search executions, data interpretations, and critical conclusions were independently conducted and verified by the human authors, who take full responsibility for the integrity and accuracy of the publication (Table 1).

## 3. Results

The initial database search yielded a total of 481 results. After removing duplicates, four independent reviewers screened the titles and abstracts for relevance. A total of 167 articles were assessed in full text for eligibility. A total of 31 sources of evidence were included. Thirteen addressed fluid and haemodynamic management, including 12 primary studies and one systematic review. Eighteen addressed AKI and renal protection, including 17 primary studies and one systematic review. The primary evidence comprised two randomised and 10 non-randomised studies in the fluid–haemodynamic domain, and two randomised and 15 non-randomised studies in the renal domain.

### 3.1. Intraoperative Physiological Changes During CRS-HIPEC

A mechanistic understanding of the phase-dependent haemodynamic and renal insults of CRS/HIPEC is fundamental to designing rational, integrated management strategies. Perioperative disturbances mainly involve haemodynamic changes (including fluid loss and bleeding), thermal stress, and hydro-electrolyte disturbances (hyponatremia, hyperglycemia and metabolic acidosis) [16,17,18,19,20].

The procedure can be divided into three temporally and physiologically distinct phases: the cytoreductive phase, the HIPEC perfusion phase, and the post-perfusion phase.

#### 3.1.1. The Cytoreductive Phase

The cytoreductive phase is characterised by extensive peritoneal stripping, multi-visceral resections, and drainage of potentially large ascitic volumes (in patients with malignant ascites). The principal fluid challenges during this phase derive from evaporative losses from large exposed serosal surfaces, intraoperative blood loss (potentially substantial with vascular adhesions), ascites drainage, redistribution into the interstitium (“third space”), and systemic vasodilation from neuraxial and systemic anaesthetic agents [16,17,18,19,20]. CRS is often associated with blood loss, and significant sloughing of the endothelial glycocalyx in patients undergoing major abdominal surgery may exacerbate the capillary leakage syndrome and vasodilatation [21,22,23].

#### 3.1.2. The HIPEC Phase

The HIPEC phase introduces qualitatively distinct haemodynamic and nephrotoxic insults that represent the most physiologically complex period of the procedure. The intraperitoneal instillation of 2–4 L of heated carrier solution at 41–43 °C induces a hyperdynamic circulatory state: peripheral vasodilation reduces systemic vascular resistance (SVR) and mean arterial pressure (MAP); reflex tachycardia and increases in cardiac index (CI), central venous pressure (CVP), and end-tidal CO_2_ ensue; and oxygen consumption and lactate production rise, reflecting hypermetabolic physiology [16,17,18,19,20]. Significant fluid shift is directly induced by hyperthermia and cytotoxic drugs, and a pro-inflammatory state induced by surgery is worsened by cytotoxic drugs [16,17,18,19,20].

The haemodynamic response typically peaks 70–80 min into the perfusion phase and resolves within 10–15 min after its completion [16,17,18,19,20].

In addition, cardiac preload may be reduced (due to increased intra-abdominal pressure—IAP-) to collapse of the inferior vena cava, particularly in closed abdominal HIPEC [16,17,18,19]. Physiological circulatory responses to whole-body hyperthermia include a large increase in cutaneous blood flow with a subsequent decrease in splanchnic blood flow, facilitating heat transfer from the body surface [16]. During HIPEC, the abdominal viscera are directly exposed to heat stress and, therefore, the remarkable heat-induced vasodilation of the splanchnic vessels occurs, resulting in greater retention of blood in the splanchnic circulation [16,18,19].

Reis et al. provided a quantification of haemodynamic changes in CRS-HIPEC, using a pulse contour system to measure CI and Stoke Volume Variation (SVV) across eight standardised time-points [24]. CI increased progressively from a mean of 2.3–2.5 L·min^−1^·m^2−1^ at the beginning of CRS to 3.4 L·min^−1^·m^2−1^ at the end of HIPEC. This increase was sustained predominantly by heart rate (HR) augmentation rather than SV, consistent with the physiological response to peripheral vasodilation and hyperthermia [24]. The increase in HR during the HIPEC phase was also described by Esteve-Pérez et al. [25]. The rise in HR is multifactorial: peripheral vasodilation with reduction in SVR, chronotropic effect of hyperthermia, and the compression of inferior vena cava with transient reduction in venous return during closed HIPEC [16,17,18,19,25].

#### 3.1.3. The Early Postoperative Period

Following CRS/HIPEC, the initial 48–72 postoperative hours are dominated by a third exudative and redistributive phase. Fluid losses via abdominal drains may reach 4–10 L per day, with drain output accounting for approximately 40% of total fluid loss, reflecting the severely wounded and exudating peritoneal surface. Progressive hypoalbuminaemia, driven by protein-rich peritoneal exudate, impairs plasma oncotic pressure and further promotes interstitial oedema. Coagulopathy is ubiquitous, driven by a combination of dilutional, consumptive, and hyperthermia-mediated platelet dysfunction mechanisms [16,17,18,19,20].

#### 3.1.4. Metabolic Parameters

Lactate increase during HIPEC is a well-recognised phenomenon, consistent with increased peripheral oxygen demand and hepatic compression [18,19,21,25]. In several patients, lactate normalised without specific treatment during the first postoperative day, indicating that the lactate elevation reflects a hyperdynamic rather than a hypoperfusion state in haemodynamically optimised patients [21,25].

The acidaemia described during CRS/HIPEC may reflect both lactic acidosis and the bicarbonate consumption associated with carbon dioxide production during hyperthermia [16,17,18,19,20,25].

### 3.2. Perioperative Fluid Management in CRS-HIPEC

**Figure 2:**
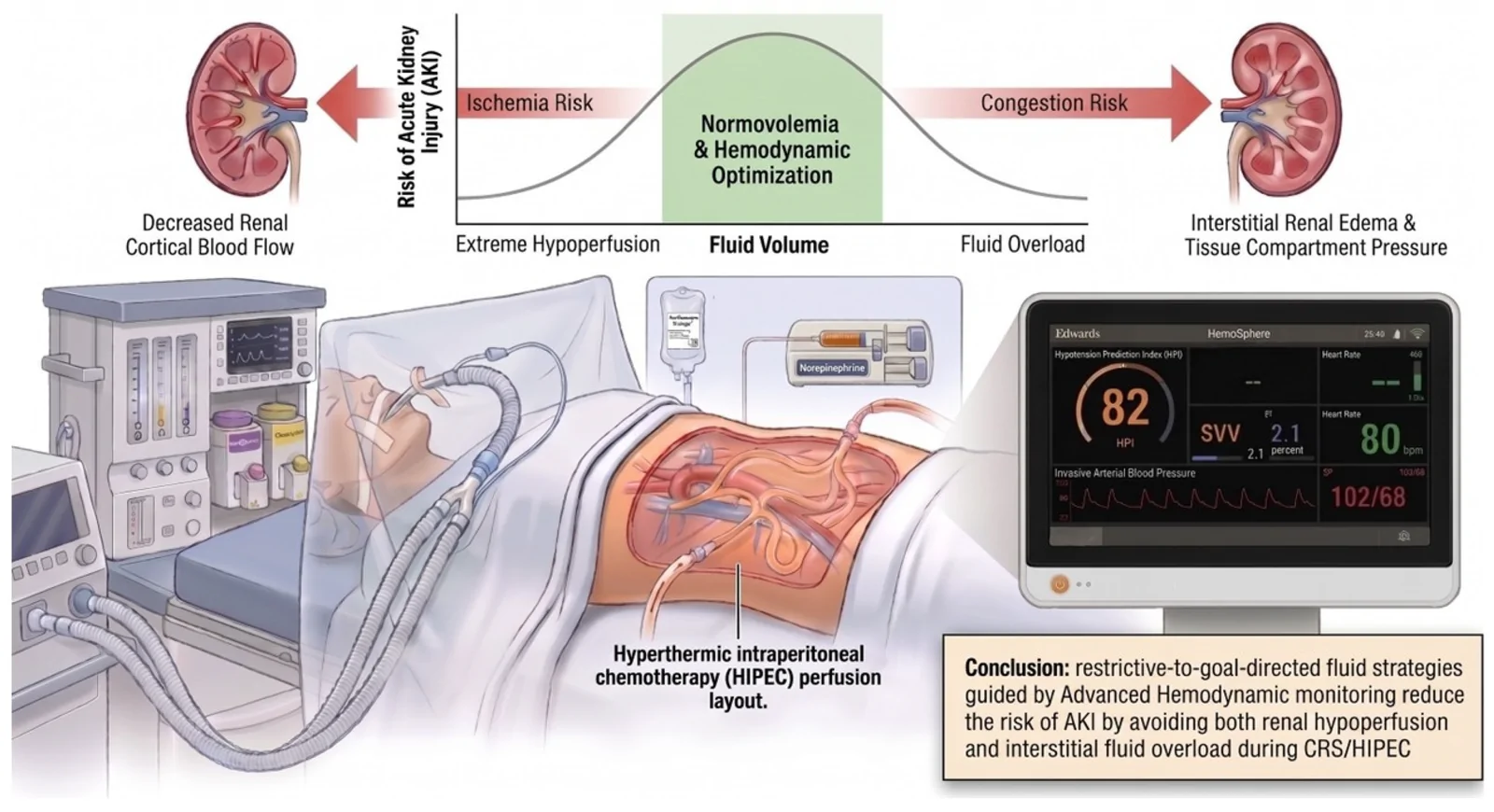
Comprehensive summary and key findings of the studies.

Management of intraoperative fluids has been the subject of much debate through the years. Early on, some authors recommended that patients be given very little fluids intraoperatively, as fluids were thought to increase the risk of postoperative complications [26,27,28,29,30,31,32]. A comprehensive summary of all included studies and their key findings is presented in Table 2 and Figure 2.

#### 3.2.1. Evidence and Strategies

The evolution of evidence in this area follows a consistent trajectory: from an era of empirical liberal administration toward increasingly restrictive and goal-directed strategies, mirroring the broader paradigm shift in perioperative medicine.

Liberal intravenous fluid administration was the historical standard for CRS/HIPEC, driven by the clinical intuition that massive evaporative losses, blood loss, and hyperthermia-induced vasodilation necessitated aggressive volume replacement. Mean intraoperative crystalloid rates of 12–16 mL·kg^−1^·h^−1^ were commonly reported in early institutional series, with total intraoperative fluid volumes frequently exceeding 10–15 L [3,36,43]. Fluid administration of 9–12 mL·kg^−1^·h^−1^ has been advocated, in particular if platinum derivatives are used, to ensure a satisfactory urinary output (at least 1 mL·kg^−1^·h^−1^) [43].

Several studies stated that this approach is harmful [25,36,40]. In a retrospective study involving 133 patients, intraoperative fluid administration at or above 15.7 mL·kg^−1^·h^−1^ was independently associated with increased incidence of complications, longer ICU stay, and greater rates of prolonged mechanical ventilation [36].

Peng et al. described (in 167 patients) high fluid rate (above the median of 7.4 mL·kg^−1^·h^−1^) as independently predictive of increased complications and longer length of stay (LOS) [40].

These results identify a consistent dose–response relationship between intraoperative fluid volume and postoperative morbidity: relevant harm thresholds include a volume ≥15.7 mL·kg^−1^·h^−1^, above-median >7.4 mL·kg^−1^·h^−1^, and >14 mL·kg^−1^·h^−1^ [25,36,40].

Fluid overload promotes interstitial oedema, impairing anastomotic healing, pulmonary gas exchange, and gastrointestinal motility [26,44]. In the context of HIPEC-phase hyperthermia, volume overload increases myocardial wall stress and oxygen demand at a time when cardiac reserve is already taxed by vasodilation-driven tachycardia [20,21]. Dilutional coagulopathy was superimposed on an already compromised haemostatic system amplifies bleeding risk [24] and, critically for renal outcomes, interstitial oedema may increase renal compartment pressure, reducing effective cortical perfusion and amplifying the nephrotoxic vulnerability established during the HIPEC phase [26,27].

#### 3.2.2. Restrictive Fluid Therapy: Safety, Feasibility, and Outcomes

Formalised restrictive fluid protocols represent the most direct response to evidence of harm from liberal administration. The core elements of these protocols are consistent across published series: a target fluid rate of 5–8 mL·kg^−1^·h^−1^; prophylactic vasopressor infusion (vasopressin 0.02 units/hour or low-dose noradrenaline) to manage vasodilation without volume loading; urine output (UOP) targets of 0.5 mL·kg^−1^·h^−1^ during cytoreduction and 1 mL·kg^−1^·h^−1^ during HIPEC; and titration to haemodynamic endpoints rather than fixed-rate infusion (Figure 2) [36,37,38].

Almerey et al. described the first structured restrictive protocol in this context, demonstrating its safety and feasibility in 35 patients with outcomes comparable or superior to published benchmarks [37]. Hendrix et al. confirmed these findings with a similar restrictive protocol, targeting fluid delivery at 500 mL·h^−1^ with more liberal use of vasopressors—demonstrating significant reductions in LOS and complication rates compared with historical controls managed without a standardised protocol and a permissive fluid management approach (1000 mL·h^−1^) [38]. The conceptual key of restrictive protocols is the deliberate uncoupling of vasodilation management from volume management: HIPEC-phase hypotension is predominantly vasodilatory in mechanism and should be addressed primarily with vasopressors than with fluid boluses, thereby avoiding the deleterious consequences of volume overload while maintaining MAP and end-organ perfusion [39].

#### 3.2.3. Goal-Directed Fluid Therapy: Principles, Evidence, and Limitations

The so-called goal-directed fluid therapy (GDFT) integrates the advantages of both liberal and restrictive approaches by replacing fixed-rate administration with dynamic, individualised titration based on real-time haemodynamic parameters of preload responsiveness [27,28,30]. In major abdominal surgery, setting GDFT within ERAS frameworks demonstrated reductions in complications, ICU stay, and time to oral intake [28,45].

The first available RCT comparing GDFT versus standard fluid management in CRS/HIPEC demonstrated significant reductions in complications and LOS in the study group, but 78.9% of GDFT patients received furosemide versus 0% of controls (*p* < 0.0001), thus introducing a pharmacological confounding that limits interpretation of the findings [34].

Esteve-Pérez et al. demonstrated 100% protocol adherence with a haemodynamic-based GDFT protocol in 92 patients, though the study was primarily descriptive [25]. Tuncel et al. reported the largest retrospective GDFT cohort to date (398 patients managed with pulse contour-derived CO monitoring), achieving a mean intraoperative fluid rate of 6.4 mL·kg^−1^·h^−1^ with acceptable morbidity and mortality [42]. Thanigaimani et al., using LiDCOrapid™ system to guide haemodynamic management, demonstrated effective maintenance of SV and MAP within pre-defined ranges [33].

On the other end, Dranichnikov et al. reported a paradoxically increased Clavien–Dindo morbidity score in their GDFT-managed cohort compared with standard management, despite shorter hospital stay [41]. The authors hypothesised that the hyperdynamic circulatory state during the HIPEC phase may generate “physiological fluid responsiveness”—defined by SVV/PPV thresholds—that prompts GDFT algorithms to administer fluid at precisely the phase when restriction would be most beneficial [41].

### 3.3. Haemodynamic Monitoring

The reliability of haemodynamic monitoring parameters is profoundly context-dependent in CRS/HIPEC. Invasive arterial blood pressure is considered essential (Figure 2) [43].

CVP has been consistently identified as unreliable in this setting: the mechanical effects of abdominal distension, increased IAP (particularly during closed HIPEC), thoracic epidural-mediated venous capacitance changes, and the hyperdynamic circulatory state all alter CVP independently of true intravascular volume [35].

CO monitoring is strongly recommended, with pulse contour analysis for calculation of SV and responsiveness indices more commonly used (SVV an PPV) [43,46]. The use of the esophageal Doppler is reported to assist in fluid assessment [47]. CO monitoring is essential to determine whether the use of vasopressors (to maintain MAP above 65 mmHg) and/or inotropic drugs (e.g., dobutamine) are needed to restore perfusion after preload optimisation. Perioperative administration of low-concentration norepinephrine is considered as a safe and effective strategy to maintain a minimum systolic blood pressure and reduce the incidence of hypotension [48,49]. Early use of low-concentration norepinephrine has been suggested as soon as arterial pressure is low, before expected effects of fluid challenges [50,51].

Initial reports show that the incorporation of new artificial intelligence-based haemodynamic monitoring tools could be a valuable help to manage hypotension and fluid therapy during CRS/HIPEC [52,53,54].

### 3.4. Acute Kidney Injury After CRS- HIPEC

**Figure 3:**
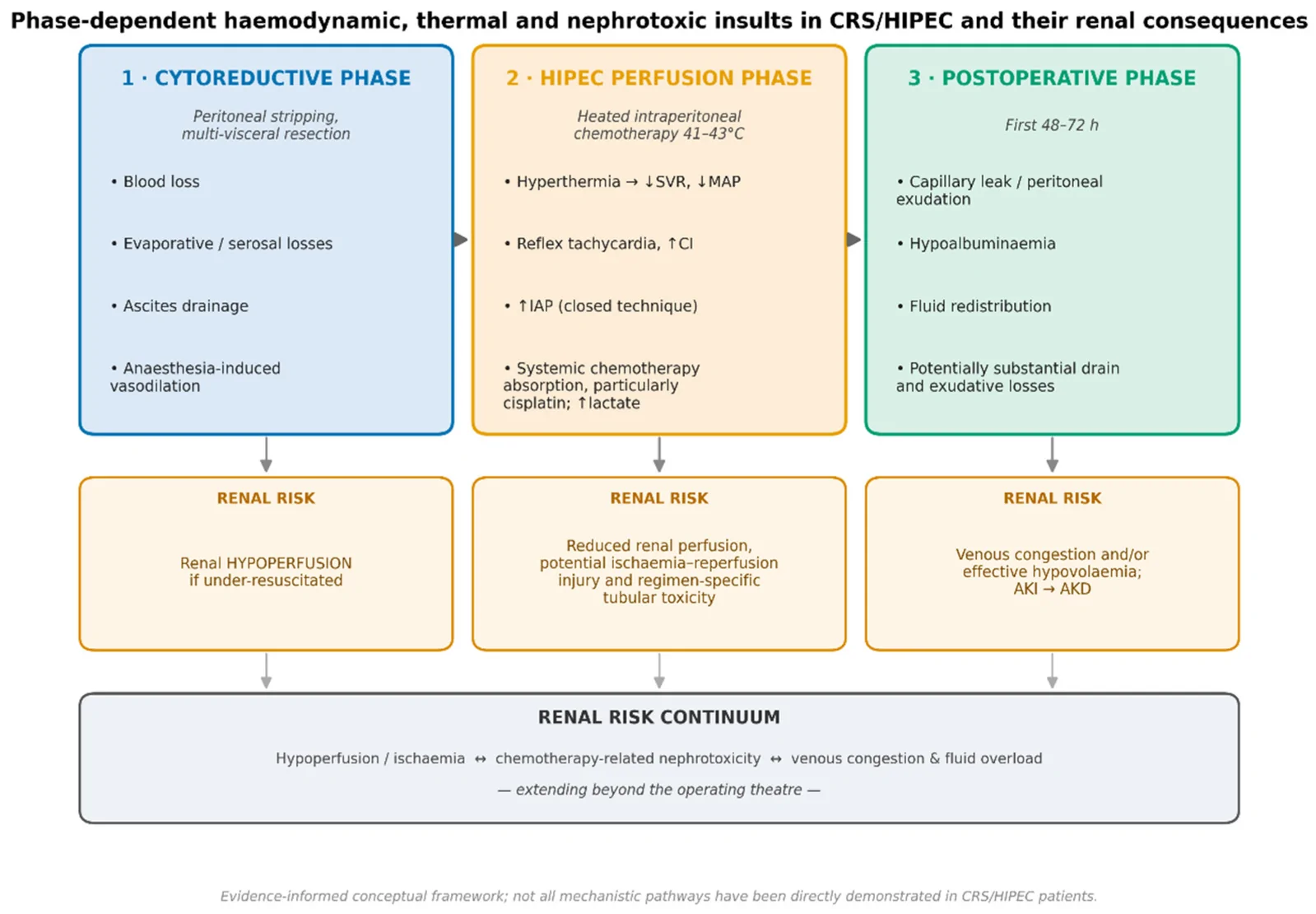
Phase-dependent haemodynamic, thermal and nephrotoxic insults of CRS/HIPEC and their renal consequences.

Acute kidney injury (AKI) is one of the most common complications after CRS + HIPEC (Figure 3) [55,56,57]. Eighteen sources of evidence addressed postoperative acute kidney injury and renal protection in patients undergoing CRS/HIPEC. These comprised 17 primary studies—two randomised controlled trial and 15 non-randomised studies—and one systematic review. The included studies differed substantially in terms of cancer type, HIPEC regimen, chemotherapeutic agent and dose, definition of renal injury, follow-up duration, and nephroprotective co-interventions. Their main characteristics and findings are summarised in Table 3.

**Figure 4:**
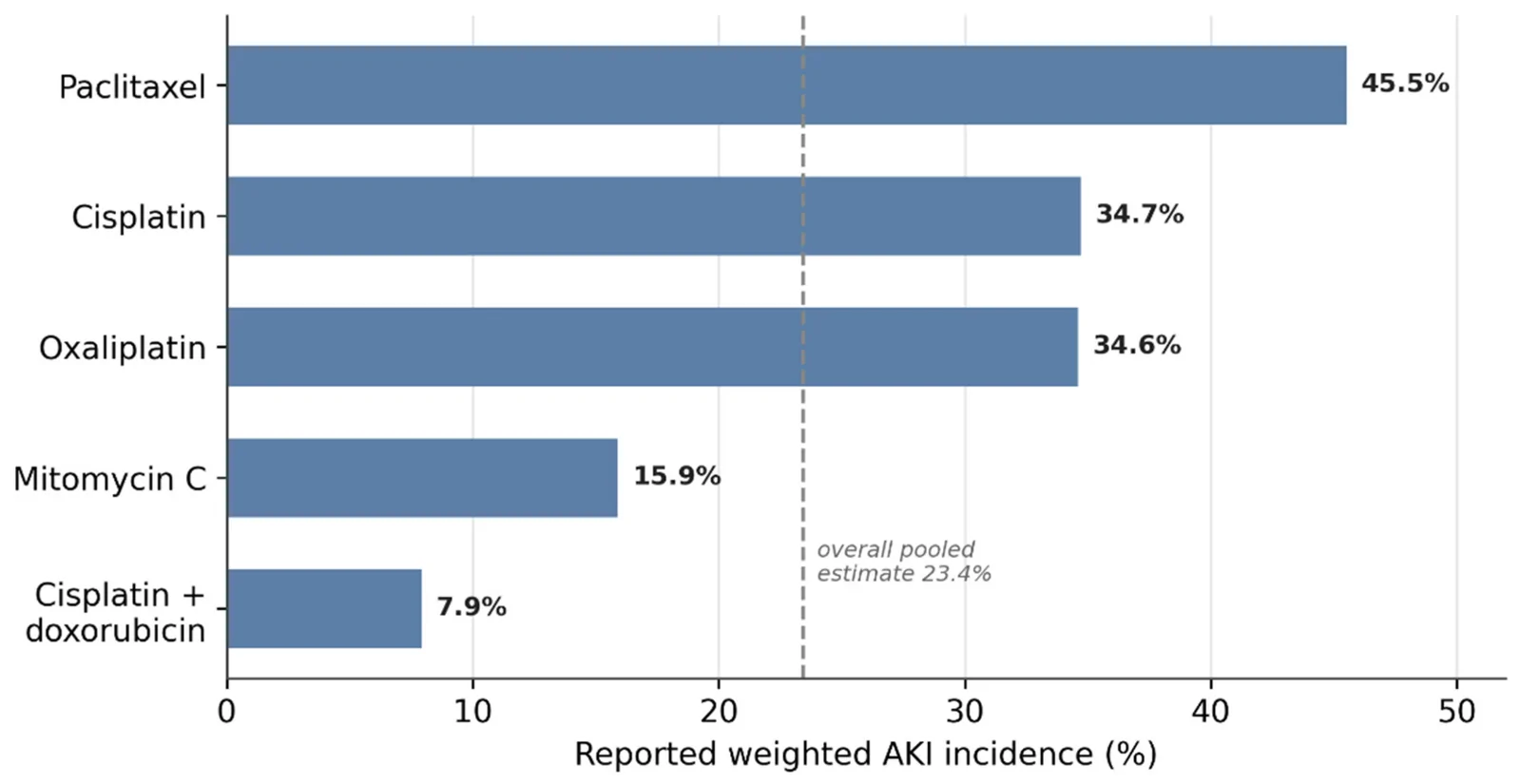
Reported weighted acute kidney injury incidence by HIPEC chemotherapy regimen, from a single systematic review [79] (Solanki et al. 2024; >1400 patients across the included studies). Weighted incidence was highest for paclitaxel (45.5%), followed by cisplatin (34.7%) and oxaliplatin (34.6%), with lower rates for mitomycin C (15.9%) and cisplatin–doxorubicin (7.9%); the dashed line marks the overall pooled estimate (23.4%) across heterogeneous regimens, not a clinical threshold. These estimates are descriptive and should not be interpreted as direct comparative measures of nephrotoxicity, because the contributing studies differed in chemotherapy dose, patient population, HIPEC technique, AKI definition and follow-up period.

Renal risk during the HIPEC phase is multifactorial and simultaneous (Figure 3) [56,73]. First, hyperthermia-induced renal vasoconstriction reduces cortical blood flow at a time when tubular metabolic demand is increased [55,56,57,73,74,75,76,77,78]. Second, direct nephrotoxic chemotherapy—particularly cisplatin and oxaliplatin—enters the systemic circulation via peritoneal absorption, exposing proximal tubular cells to cytotoxic drug concentrations (Figure 4) [10,57,79]. Cisplatin nephrotoxicity is mediated through oxidative stress, mitochondrial dysfunction, DNA damage, and activation of tubular apoptosis pathways; it is dose-dependent and cumulative, with single-agent doses above 75–100 mg/m^2^ associated with substantially higher AKI incidence [57]. Third, haemodynamic instability and reduced effective renal perfusion pressure during this phase create an ischaemia–reperfusion pattern of injury that potentiates direct tubular toxicity [10,28,31]. Intraoperative hyperthermia has been identified as an independent AKI risk factor [56,74].

Renal function typically continues to decline in the early postoperative days before recovering, following a trajectory that reflects the combined insults of intraoperative ischaemia, chemotoxicity, and subsequent haemodynamic management [73]. The transition from AKI to acute kidney disease (AKD—defined as renal dysfunction persisting beyond 7 days) has been described as a distinct and prognostically significant outcome in this population, associated with preoperative ascites and systemic inflammatory activation [57].

#### 3.4.1. Incidence and Definitions: A Problem of Heterogeneity

The available literature is characterised by marked heterogeneity in AKI definitions, chemotherapy regimens, patient populations, and follow-up periods—a heterogeneity that substantially complicates direct comparison of incidence estimates and risk factor analyses.

The reported incidence of AKI following CRS/HIPEC varies markedly across the included literature, ranging from 7.9% to 45.5% [58,79]. This wide variability reflects the combined effects of differences in AKI definition criteria, chemotherapy agents and doses, patient populations, and follow-up duration rather than true biological heterogeneity.

Arjona-Sánchez et al. directly compared RIFLE and AKIN criteria in the same cohort of 141 patients and identified substantially higher AKI rates with RIFLE (30.5%) than AKIN (22%) criteria (*p* < 0.001), with RIFLE demonstrating superior discriminative capacity [60]. The systematic review by Solanki et al., encompassing 16 studies with data from over 1400 patients, reported an overall weighted AKI incidence of 23.4%, with only 1.78% of patients requiring renal replacement therapy (RRT), suggesting that most AKI events are functional and recoverable rather than severe and irreversible [79].

Kapoor et al. in a laparoscopic HIPEC population with high-dose cisplatin (mean 106.6 mg/BSA) recorded zero AKI cases, yet documented significant electrolyte disturbances (hypocalcaemia 94%, hypophosphataemia 84%), underscoring that subclinical tubular perturbations can occur in the absence of creatinine-based AKI thresholds [63].

#### 3.4.2. Risk Factors and Risk Stratification

A consistent set of AKI risk factors emerges across the included studies. Cisplatin-based HIPEC regimens are the most frequently identified risk factor. The nephrotoxic risk is dose-dependent: high-dose cisplatin (>75–100 mg·m^2−1^) carries substantially higher AKI risk than low-dose regimens (3.7% AKI with 50 mg·m^2−1^ cisplatin, 34.7% overall with cisplatin-HIPEC) [58,79]. Oxaliplatin-based regimens carry a comparable overall incidence of 34.6% [79].

Patient-level risk factors consistently associated with AKI across multiple studies include: older age, pre-existing renal dysfunction (reduced glomerular filtration ratio—eGFR-), hypertension, diabetes mellitus, preoperative ascites (OR = 3.501), low serum albumin, perioperative blood transfusion, shorter interval between preoperative carboplatin chemotherapy and surgery, and perioperative use of nephrotoxic co-medications [57]. Intraoperative factors include excessive blood loss, prolonged operative duration, intraoperative hypotension duration (OR = 1.030), and intraoperative hyperthermia (OR = 1.36) [57]. Notably, while baseline lifestyle factors such as chronic alcohol consumption are recognised contributors to subclinical endothelial and renal vascular vulnerability, they remain largely unmeasured and underreported across the current CRS/HIPEC literature, representing a potential unmeasured confounder in retrospective risk stratification.

An important and previously underappreciated risk factor is the choice of HIPEC perfusate: Chen et al. identified peritoneal dialysis solution as an independent risk factor for acute renal impairment, an effect attributed to dextrose absorption-induced hyperosmolarity and oxidative tubular stress [65].

Krause et al. developed a preoperative AKI risk prediction model using electronic health record data (N = 412, AKI incidence 8.7%), achieving an AUC of 0.82 with a final model incorporating BMI, haemoglobin, male sex, and total intraperitoneal cisplatin dose [69]. Synthetic minority oversampling (SMOTE) was applied to address class imbalance. The model has been internally validated only—external prospective validation in independent multicentre cohorts is required before clinical implementation [69].

Cheng et al. reported a significant reduction in glomerular filtrate in the short-term following cisplatin-HIPEC, with partial recovery in the longer term [70]. CKD was documented in 30.9% (17/55) of patients [77]. Critically, this study identified parecoxib as an independent risk factor for both AKI and CKD development [70].

Sin et al. described AKI incidence and risk factors after CRS/HIPEC with cisplatin for ovarian cancer 100 mg/m^2^ in 47 patients. AKI incidence was 40.4% (according to NCI-CTCAE 3.0), and grade 3–4 in 10.6% [61].

#### 3.4.3. Nephroprotective Strategies in CRS/HIPEC

**Figure 5:**
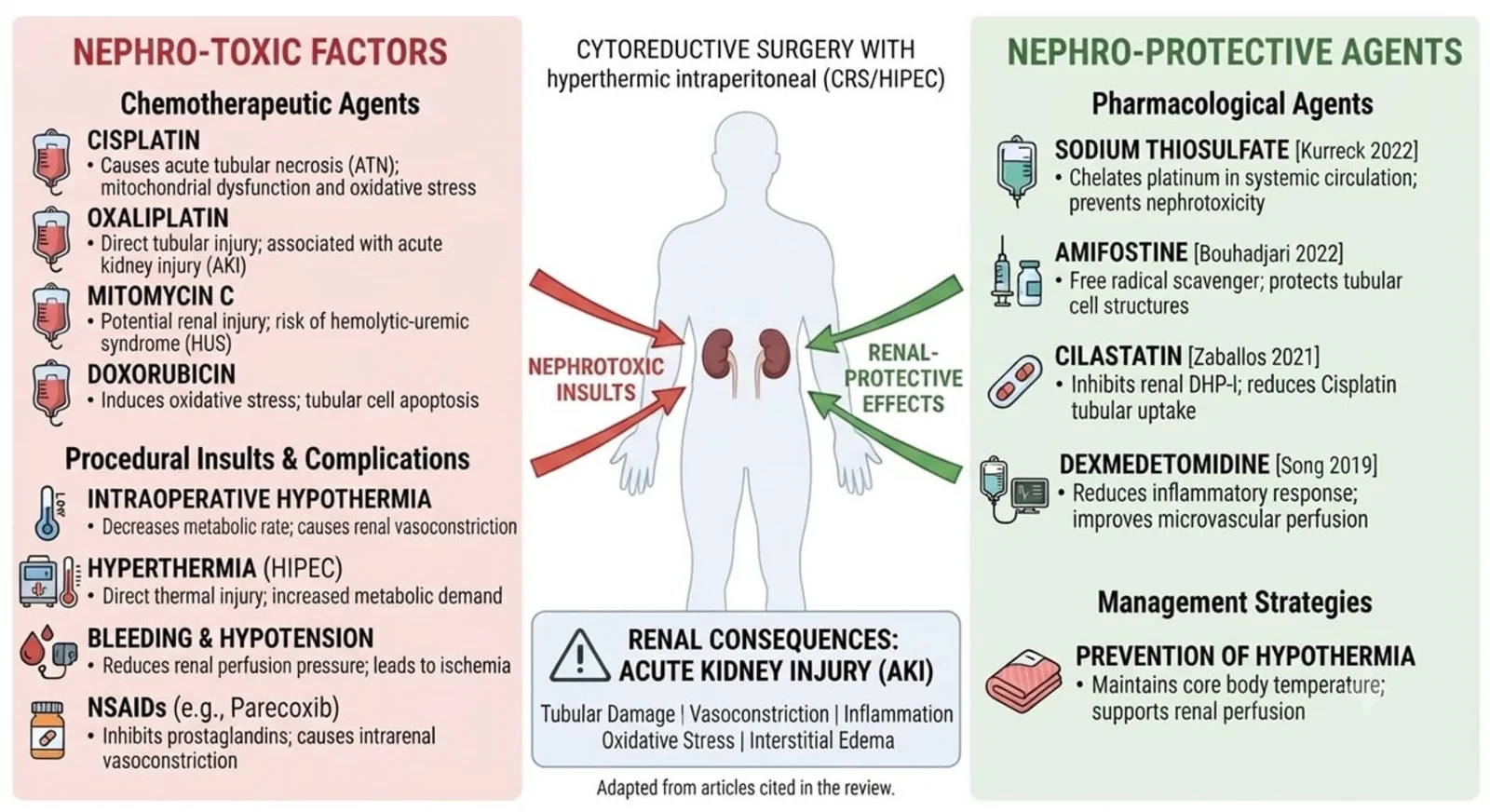
Strategies to mitigate AKI risk in CRS/HIPEC [59,62,67,68].

Pharmacological and non-pharmacological strategies to mitigate AKI risk in CRS/HIPEC have been evaluated in a limited number of studies, predominantly retrospective. Four pharmacological agents—sodium thiosulfate (STS), amifostine, cilastatin, and dexmedetomidine—have been specifically investigated in this context (Figure 4 and Figure 5, Table 3).

### 3.5. Sodium Thiosulfate

STS infusion at 9 gr/m^2^ prior to HIPEC and at 12 gr/m^2^ at the end of the procedure acts as a cisplatin-selective chelating agent, binding free cisplatin in the systemic circulation and urine while sparing intraperitoneal concentrations at the tumour site—a pharmacokinetic selectivity that makes it uniquely well-suited as an adjuvant nephroprotective agent in the HIPEC context [79,80]. However, the clinical safety profile of STS warrants careful consideration; its hyperosmolar sodium load can lead to hypernatremia and metabolic acidosis, requiring extended post-operative monitoring in an intensive care setting.

Laplace et al. assessed the impact STS in preventing AKI following HIPEC with cisplatin with a before–after study, comparing a period without STS and a subsequent one with STS. STS treatment reduced the incidence of AKI (defined as postoperative serum creatinine >1.6 times elevation of baseline value) to 0 in 38 patients, compared to an incidence of 11/35 patients (31.4%) reported without STS (*p*  <  0.005) [64].

Glennon et al., in a retrospective case-controlled study, investigated 30 patients who had interval cisplatin-based CRS/HIPEC. Seven received cisplatin 50 mg/m^2^ without the addition of STS, and 23 patients received cisplatin 100 mg/m^2^ and STS. One episode of AKI was registered after cisplatin 50 mg/m^2^ without STS, and this was sustained at three months. No patients within the STS cohort sustained a renal injury [66].

In a study by Kurreck et al., 46 patients treated with STS vs. a historical control of 192 patients demonstrated a significant reduction in AKI incidence after exposure to cisplatin 75 mg/m^2^, from 30.7% to 6.5% (*p* = 0.001), with no patient in the STS group requiring RRT vs. 6 (3.1%) in the historical control group (*p* = 0.225, NS) [68]. On multivariable analysis, STS was independently protective (OR = 0.089, *p* = 0.001) [68].

In a retrospective analysis, Vachez et al. treated 220 patients curatively for peritoneal surface malignancy with cisplatin-based CRS/HIPEC and categorised three groups based on the management of cisplatin-induced nephrotoxicity (preoperative hyperhydration alone, preoperative hyperhydration plus STS, STS alone) [81]. Mean serum creatinine and incidence of AKI were significantly higher in the group without STS. The authors concluded that there is no need for preoperative hyperhydration when STS is used to prevent cisplatin-induced nephrotoxicity [81].

Lee et al. categorised 180 patients with advanced ovarian cancer who underwent interval debulking surgery with CRS/HIPEC into two groups: STS and hydration along with acetylcysteine and mannitol [72]. In the STS group, 14 patients developed stage I AKI [72]. In the hydration group, nine patients developed AKI, including six with stage I, two with stage II, and one with stage III. The incidence of AKI did not differ significantly between the two groups [72]. Univariate and multivariate analyses identified estimated blood loss as a potential contributor to AKI occurrence (*p* = 0.046) [72].

### 3.6. Amifostine

Amifostine is an organic thiophosphate that acts as a broadly cytoprotective free radical scavenger, selectively accumulated in normal tissues relative to tumour cells due to differential alkaline phosphatase activity [79,82]. Bouhadjari et al. demonstrated in a retrospective cohort that amifostine administration significantly reduced the incidence of severe renal impairment (CrCl < 30 mL·min^−1^) from 33% (no amifostine) to 13% (amifostine; *p* = 0.04), with no patients in either group requiring dialysis [59].

### 3.7. Cilastatin

Cilastatin is a specific inhibitor of dehydropeptidase-I (DHP-I), the renal brush-border enzyme responsible for the proximal tubular uptake and conversion of imipenem. When co-administered with cisplatin, cilastatin inhibits the tubular uptake of cisplatin-derived platinum complexes, thereby reducing intracellular platinum accumulation in proximal tubular cells and attenuating cisplatin nephrotoxicity [83,84]. Zaballos et al. demonstrated in a combined retrospective/prospective study that cilastatin co-administration was associated with fewer major postoperative complications (CD 3–4), shorter ICU stay (*p* = 0.02), and shorter hospital stay (*p* = 0.005), with a significant reduction in postoperative creatinine elevation [67]. The dual anti-inflammatory and direct tubular-protective mechanism of cilastatin makes it a promising agent for this indication, and ongoing prospective evaluation is warranted.

### 3.8. Dexmedetomidine

Dexmedetomidine, a selective α_2_-adrenergic agonist with sedative, analgesic, and sympatholytic properties, has been proposed as a nephroprotective agent through multiple mechanisms: reduction in renal sympathetic tone, attenuation of ischaemia–reperfusion injury, anti-inflammatory cytokine modulation, and direct anti-apoptotic effects on tubular cells [85,86]. Song et al. randomised 38 patients to dexmedetomidine or placebo during mitomycin-HIPEC, showing a significant reduction in biomarkers of tubular injury in the dexmedetomidine group but with similar incidence of AKI by KDIGO criteria [62].

### 3.9. Non-Pharmacological Strategies

Beyond pharmacological intervention, several non-pharmacological strategies emerge from the included literature as clinically actionable nephroprotective measures. Active thermal management to prevent intraoperative hyperthermia, maintaining core body temperature below 38.5–39 °C through cooling blankets, cold intravenous fluid during the HIPEC phase, and ambient temperature control, is supported by the reported association between hyperthermia magnitude and AKI risk [56]. Cisplatin dose optimisation, adjusting total peritoneal cisplatin dose based on preoperative risk stratification, may reduce AKI incidence in high-risk patients without compromising oncological efficacy [65]. Maintenance of adequate renal perfusion pressure (MAP ≥ 65–70 mmHg) through vasopressor support during the HIPEC phase, avoidance of nephrotoxic co-medications, and adequate perioperative hydration sufficient to maintain UOP ≥ 1 mL/kg/h during HIPEC complement pharmacological strategies as part of a bundled renoprotective approach [71]. Similarly, while forced diuresis with furosemide or mannitol facilitates tubular drug clearance, it carries an inherent risk of unrecognised hypovolemia and secondary renal hypoperfusion if fluid replacement is not rigidly matched in real time. Finally, aggressive preoperative nutritional optimisation to correct hypoalbuminaemia (a consistent independent AKI risk factor in this literature) represents a modifiable preoperative target [87].

## 4. Discussion

The present review synthesises evidence from 31 studies across two mechanistically related but clinically distinct domains, arriving at several convergent themes that have direct implications for the perioperative management of CRS/HIPEC.

### 4.1. The Fluid-Kidney Nexus: An Integrated Perspective

The mechanistic interaction between fluid therapy and AKI risk in CRS/HIPEC is governed by a delicate physiological balance. Fluid overload directly contributes to interstitial oedema, which increases renal subcapsular pressure and leads to renal venous congestion. This phenomenon, exacerbated by the increased intra-abdominal pressure during the HIPEC phase, critically reduces the trans-renal perfusion gradient, impairing drug clearance and compounding cisplatin-induced tubular toxicity. Conversely, excessive fluid restriction without adequate haemodynamic monitoring may lead to unrecognised hypovolemia. In the context of hyperthermia-induced vasoplegia, this hypovolemia translates into profound renal hypoperfusion and ischemic-reperfusion injury. Therefore, optimising fluid therapy is not merely a haemodynamic goal, but a primary nephroprotective intervention.

A direct comparison between restrictive and goal-directed fluid therapy reveals that both strategies aim to prevent the morbidity of fluid overload, but through different conceptual approaches. Restrictive strategies (typically targeting 5–8 mL·kg^−1^·h^−1^) achieve this by uncoupling vasodilation management from volume resuscitation, relying on early vasopressor use rather than fluid boluses. GDFT, while dynamically tailoring fluid administration to the patient’s physiological responsiveness, carries the risk of misinterpreting the hyperdynamic, vasodilatory HIPEC phase as a state of true hypovolemia, potentially triggering inappropriate fluid boluses. To mitigate this, merging GDFT principles with an upper restrictive limit is advisable. The literature consistently identifies critical harm thresholds: fluid administration rates exceeding 15.7 mL·kg^−1^·h^−1^, and in some cohorts even >7.4 mL·kg^−1^·h^−1^, are independently associated with severe postoperative morbidity, longer ICU stays, and an elevated risk of AKI.

Within a goal-directed framework and at constant net fluid balance, actively augmenting intraoperative UO with diuretics and matched replacement reduced both AKI and overall morbidity, indicating that renal clearance of nephrotoxic drug, and not volume status alone, is a modifiable determinant of renal outcome in cisplatin-based HIPEC [71].

This integrated perspective is supported by the convergent findings of the included literature: the same parameters that define optimal fluid management (adequate MAP, CI, and UOP guided by dynamic indices) also constitute the operational targets for renal protection. The artificial separation of these two clinical domains in the existing literature, reflected in the predominance of studies addressing only one domain, may have contributed to suboptimal management protocols that optimise for either fluid balance or renal outcome without explicitly co-designing for both.

#### Clinical Implications and Take-Home Message

For practical clinical management during CRS/HIPEC, the current evidence supports a bundled, protocolized approach. First, hemodynamic monitoring should routinely incorporate continuous invasive arterial pressure alongside dynamic indices (such as SV, SVV, or PPV) using minimally invasive cardiac output monitors. Second, fluid administration should be targeted within a restrictive range (5–8 mL·kg^−1^·h^−1^), prioritising the early initiation of vasopressor infusions over recurrent fluid boluses to counteract HIPEC-induced vasoplegia. Finally, for patients receiving nephrotoxic regimens such as cisplatin-based HIPEC, pre-emptive nephroprotective strategies are mandatory. This includes individualised dose adjustments, meticulous temperature control, ensuring continuous urine output (>1 mL·kg^−1^·h^−1^) through strictly matched hydration, and the routine integration of sodium thiosulfate (STS) into institutional protocols.

### 4.2. Synthesis and Patterns of the Evidence Base

Despite the predominantly retrospective nature of the included studies, the direction of evidence across both thematic domains is notable for its consistency. For fluid therapy, the association between liberal fluid administration and increased morbidity has been replicated across multiple independent cohorts, different institutional settings, and different analytical approaches [25,26,27,28,29,30,31,32,33,34,35,36,37,38,39,40,41,42,43,44,45,46]. For AKI, the role of cisplatin dose, patient-level risk factors, and the potential benefit of specific nephroprotective agents converges across populations from different countries and surgical systems [55,56,57,58,60,61,63,64,65,66,68,69,70,73,74,75,76,77,78,79,80]. This consistency strengthens the confidence with which pragmatic clinical recommendations can be drawn, even in the absence of high-quality randomised evidence.

### 4.3. Limitations of the Current Evidence

Several important limitations must be acknowledged. First, the most significant gap is the absence of adequately powered, prospective randomised trials specifically designed to compare fluid management strategies in CRS/HIPEC with pre-specified renal outcome endpoints. The first of the only two RCTs identified addressing fluid-haemodynamic management focused on IAP technique rather than fluid strategy per se and enrolled only 33 patients [24]. The second available RCT compared GDFT versus standard fluid management, but 78.9% of GDFT patients received furosemide versus 0% of controls [34]. The only nephroprotective RCT enrolled 38 patients and was powered for the primary endpoint of creatinine clearance change rather than AKI incidence [62].

Moreover, a limitation of current GDFT approaches in HIPEC is the poor validation of the standard dynamic indices of fluid responsiveness during the hyperdynamic, elevated-IAP, tachycardic conditions prevalent during the hyperthermic perfusion phase [41]. Additionally, while our review protocol was prospectively designed, formal public registration on the Open Science Framework occurred retrospectively, which represents a methodological limitation.

Second, the definition of key exposures and outcomes varies substantially across studies. “Restrictive” and “liberal” fluid therapy are defined inconsistently—ranging from absolute volume thresholds to median splits of institutional cohorts—making direct quantitative comparisons across studies unreliable. Similarly, AKI definitions using RIFLE, AKIN, KDIGO, or NCI-CTCAE criteria identify overlapping but non-identical patient populations [61,79].

Third, all four nephroprotective pharmacological agents with supporting evidence in CRS/HIPEC (STS, amifostine, cilastatin, and dexmedetomidine) have been evaluated only in retrospective cohorts (with the exception of dexmedetomidine’s small RCT by Song), limiting causal inference. None has been evaluated in a multicentre, prospective randomised trial adequately powered for clinical AKI endpoints.

Fourth, important confounders—including the specific chemotherapy agent and dose, technique (open vs. laparoscopic, open vs. closed HIPEC), duration of HIPEC, and extent of cytoreduction—are inconsistently reported or adjusted for across studies, limiting the generalisability of individual findings.

A further limitation concerns the timing of protocol registration. Because the project was initially conceived as a narrative synthesis and was converted to a formal scoping review during screening, the protocol was deposited on the Open Science Framework after the initial searches had been executed rather than before. Retrospective deposition does not constrain post hoc methodological choices as reliably as prospective registration, and our findings should be interpreted with that caveat.

Finally, in accordance with the JBI methodology for scoping reviews and the PRISMA-ScR framework, we deliberately did not perform a formal methodological quality appraisal or risk-of-bias assessment of the included studies. The fundamental objective of this scoping review was to map the breadth of the available evidence and identify conceptual gaps regarding fluid management and AKI, rather than to evaluate the certainty of the evidence to inform strict clinical guidelines. Consequently, the clinical recommendations derived from this synthesis should be interpreted with caution, acknowledging the unassessed risk of bias and the predominantly retrospective nature of the primary literature.

### 4.4. Future Research Priorities

The evidence gaps identified by this review point to several high-priority research directions. Most urgently, prospective randomised trials are needed to compare standardised restrictive versus GDFT fluid protocols in CRS/HIPEC, with pre-specified co-primary endpoints encompassing both morbidity outcomes (Charlson Comorbidity Index, LOS) and renal endpoints (AKI incidence, urinary biomarkers, eGFR trajectory). Such trials should stratify by chemotherapy regimen (cisplatin vs. non-cisplatin) and PCI score, and include standardised haemodynamic monitoring protocols to enable assessment of the added value of advanced monitoring modalities.

For nephroprotection, multicentre randomised trials of STS and amifostine in cisplatin-based HIPEC are the most clinically pressing research priority, given the magnitude of the effects observed in retrospective cohorts. A factorial trial design incorporating both fluid strategy and nephroprotective pharmacotherapy as randomised factors would enable assessment of interaction effects between these two intervention domains. The Krause et al. AKI risk prediction model warrants prospective external validation in independent multicentre cohorts and refinement with prospectively collected biomarker data to enable real-time perioperative risk stratification [69].

The trial of Gao et al. provides both a template and an immediate priority: multicentre replication of urine-guided hydration in histologies other than pseudomyxoma peritonei and in non-cisplatin regimens, with AKI adjudicated by creatinine- and biomarker-based criteria in order to exclude the outcome-measurement circularity inherent in a urine-output-targeted intervention [71].

Finally, the long-term renal consequences of CRS/HIPEC—including CKD progression and its impact on eligibility for repeat procedures or adjuvant systemic chemotherapy—represent an underexplored but clinically important outcome domain that should be systematically incorporated into long-term follow-up registries.

## 5. Conclusions

Perioperative fluid management and renal protection represent two mechanistically interdependent challenges during CRS/HIPEC. The available evidence, though predominantly observational, supports a consistent pattern of clinical observation.

Fluid therapy: Liberal intraoperative fluid administration has been consistently associated with increased postoperative morbidity and prolonged hospital stay in observational cohorts, favouring restrictive-to-goal-directed strategies.

Target intraoperative fluid rates of 5–8 mL·kg^−1^·h^−1^, guided by dynamic haemodynamic parameters from minimally invasive CO monitors, represent the current evidence-based approach. Vasopressor support is the appropriate primary response to HIPEC-phase vasodilatory hypotension. Central venous pressure is an unreliable preload guide in this context and should not be used as the sole fluid titration endpoint.

Renal protection: AKI incidence in CRS/HIPEC ranges from under 10% to over 30% depending on cisplatin dose and patient risk profile. Preoperative risk stratification using validated predictive tools should guide individualised cisplatin dose adjustment and prophylactic nephroprotective strategy selection in high-risk patients. STS has the strongest retrospective evidence for AKI prevention in cisplatin-HIPEC and represents a promising candidate for bundled nephroprotective protocols, although definitive clinical confirmation in prospective randomised trials is still needed. Amifostine and cilastatin are promising alternatives warranting prospective evaluation. Active thermal management to prevent intraoperative hyperthermia is an underappreciated but evidence-supported non-pharmacological nephroprotective measure. Prospective randomised trials integrating standardised fluid protocols and renal outcome endpoints remain necessary to establish causal guidance.

## Figures and Tables

**Table 1:** Search strategies and keywords.

Criteria	Details
Databases searched	PubMed/Medline, the Cochrane Central Register of Controlled Clinical Trials, ScienceDirect, EMBASE, and Scopus
Search period and language	1 January 2014–31 December 2025, English
Article types	Primary research articles, systematic reviews, and meta-analyses
Primary keywords and MeSH terms	Cytoreductive surgery, HIPEC, hyperthermic intraperitoneal chemotherapy, anaesthetic management, haemodynamic monitoring, goal-directed therapy, acute kidney injury, fluid therapy, intraoperative complications, perioperative and postoperative complications.

MeSH = Medical Subject Headings.

**Table 2:** Monitoring and haemodynamic targets in the primary fluid-management studies.

Study, Year	Number of Patients	Monitoring Modality	Parameters Used	Study Design/Haemodynamic Strategy	Key Findings
Thanigaimani, 2014 [33]	25	LiDCOrapid (pulse-power)	CO	Prospective observational; GDFT (HIPEC phase)	No significant change in HR and CO. SVR dropped during the HIPEC phase. Average volume of fluid given was 748 mL in the first 30 min and 630 mL in the second 30 min with average urine output of 307 and 445 mL.
Colantonio, 2015 [34]	80	FloTrac sensor, Vigileo platform (pulse-contour)	CI, SVV	RCT; GDFT	Incidence of major abdominal complications was lower in GDFT group (10.5% vs. 38.1%). Median LOS shorter in GDFT group (19 vs. 29 days). GDFT group received a significantly lower amount of total fluid and crystalloids.
Redondo, 2017 [35]	18	PiCCO (transpulmonary thermodilution, pulse-contour)	GEDV, CI	Pilot; preload-index comparison	No significant haemodynamic measurements changes (CI, GEDV, SVV) across phases. No correlation between CVP and GEDV. GEDV values may be more appropriate for monitoring cardiac preload.
Eng, 2017 [36]	133	Not standardised	Not protocolised	Retrospective	Intraoperative fluid rate was significantly associated with morbidity. Patients receiving > 15,7 mL·kg^−1^·h^−1^ experienced a 43% increase in complication rate.
Almerey, 2018 [37]	35	Standard monitoring (no advanced CO monitor)	MAP, urine output	Retrospective; restrictive	Restrictive protocol resulted in Clavien–Dindo grade 3–4 events in 5 patients. Median hospital stay was 7 days with no deaths at 30 days. Fluid restriction appears to be safe and feasible.
Esteve-Pérez, 2019 [25]	92	FloTrac sensor, Vigileo platform (pulse-contour)	SVV, CI, MAP	Prospective observational; GDFT	Great variability in intraoperative fluid therapy needs. SVV monitoring enables individual adjustment of fluid therapy. Direct relationship observed between peritoneal carcinomatosis index and surgery duration, fluid therapy, and transfusion.
Hendrix, 2019 [38]	169	Standard monitoring (no advanced CO monitor)	MAP, vasopressor use	Retrospective; restrictive	LOS reduced from 11.5 to 9.7 days in restrictive group (*p* < 0.01). Incidence of any 60-day complication decreased from 45% to 28% in restrictive group (*p* = 0.02).
Reis, 2020 [24]	33	FloTrac sensor, EV1000 platform (pulse-contour, ScvO_2_)	CI, SVV, ScvO_2_	RCT; GDFT-managed	No difference in haemodynamic parameters between low and high IAP groups, except CVP which was higher in high IAP group. High IAP (18–22 mmHg) was well tolerated with no haemodynamic instability.
Kim, 2021 [39]	20	FloTrac sensor, EV1000 platform	CI, SVV	Prospective observational; advanced monitoring	HIPEC increases body temperature and cardiac output and decreases systemic vascular resistance. Transpulmonary thermodilution approach is helpful for intraoperative management.
Peng, 2021 [40]	167	Not standardised	Not protocolised	Retrospective	Low-IVF group had less blood loss, less need for vasopressors, and fewer major complications (14.5% vs. 33.3%). Higher intraoperative fluid intake associated with increased LOS.
Dranichnikov, 2023 [41]	509	NR	NR	Retrospective; GDFT vs. standard	Overall postoperative morbidity Grade III-V was higher in the GDFT group (30% vs. 22%). Mean length of stay was shorter in the GDFT group (17 vs. 26 days). No correlation between fluid management and postoperative haemorrhage.
Tuncel, 2024 [42]	398	NR	NR	Retrospective observational; GDFT	Mean intraoperative fluid rate was 6.4 mL·kg^−1^·h^−1^ with 16.3% experiencing Clavien–Dindo Grade 3–4 events. GDFT with advanced monitoring devices is a successful alternative to traditional fluid management.

Monitoring and haemodynamic targets in the primary fluid-management studies (*n* = 12). Some rows describe protocols or titration targets rather than a discrete device. FloTrac is the pulse-contour sensor/algorithm, whereas Vigileo and EV1000 are the associated monitoring platforms. CO: cardiac output; CI: cardiac index; SVV: stroke volume variation; ScvO_2_: central venous oxygen saturation; MAP: mean arterial pressure; GEDV: global end-diastolic volume; GDFT: goal-directed fluid therapy; RCT: randomised controlled trial; LOS: length of stay; IAP: intra-abdominal pressure; NR: not reported.

**Table 3:** Main characteristics and findings of the included studies addressing postoperative acute kidney injury.

Author, Year (Journal)	Design	Number of Patients	Exposure/Intervention	Key Findings
Hakeam, 2014 [58]	Retrospective	53	Cisplatin nephrotoxicity—HIPEC with cisplatin 50 mg/m^2^ + doxorubicin.	AKI incidence (KDIGO/RIFLE): 3.7%. Hypomagnesaemia significantly elevated at day 7 and day 30. One AKI patient progressed to CKD. Low-dose cisplatin substantially reduces nephrotoxicity risk.
Bouhadjari, 2015 [59]	Retrospective	52	Amifostine 910 mg/m^2^ iv vs. no amifostine—cisplatin-HIPEC.	Severe renal impairment (CrCl < 30 mL·min^−1^) within 4 postoperative days: 13% (amifostine) vs. 33% (no amifostine) (*p* = 0.04). No patient in either group required dialysis.
Arjona-Sánchez, 2016 [60]	Retrospective cohort	141	RIFLE vs. AKIN criteria for acute renal dysfunction—HIPEC for ovarian and non-ovarian.	AKI incidence: 30.5% (RIFLE) vs. 22% (AKIN) (*p* = 0.001 RIFLE superiority principally at less severe levels). No patient required RRT.
Sin, 2017 [61]	Retrospective cohort	47	AKI incidence and risk factors—ovarian cancer CRS/HIPEC with cisplatin 100 mg/m^2^.	AKI incidence: 40.4% (NCI-CTCAE 3.0); grade 3–4 in 10.6%. Long-term dialysis: 4.3%. Risk factors: older age, poorer baseline renal function, shorter interval from carboplatin to surgery, perioperative blood transfusion.
Song, 2019 [62]	RCT (double-blind, placebo-controlled)	38	HIPEC with mitomycin Dexmedetomidine 1 mcg·kg^−1^ bolus + 0.5 mcg·kg^−1^·h^−1^ infusion vs. placebo.	Lowest CrCl absolute change: not significant (*p* = 0.082). Percentage of reduction in CrCl significantly lower (*p* = 0.037) in study group. AKI incidence similar (all KDIGO stage 1). ICU stay shorter (*p* = 0.034).
Kapoor, 2019 [63]	Retrospective cohort	23	Laparoscopic HIPEC with high-dose cisplatin (mean 106.6 mg/BSA).	AKI criteria: NCI-CTCAE v5.0. No AKI cases recorded. Median Cr change in 2nd postoperative day +0.09 mg/dL. Electrolyte disturbances occurred despite absence of overt AKI.
Laplace, 2020 [64]	Before-and-after non-randomised study	73	Cisplatin-based HIPEC; STS versus no STS.	AKI occurred in 0/38 patients receiving STS versus 11/35 patients without STS (31.4%; *p* < 0.005).
Chen, 2020 [65]	Retrospective cohort	169	Risk factors for acute renal impairment post-CRS/HIPEC (ARI defined by NCI-CTCAE v5.0 criteria).	NCI-CTCAE v5.0 criteria ARI incidence 12.4%. Independent multivariable predictors cisplatin HIPEC regimen and peritoneal dialysis solution as HIPEC perfusate.
Glennon, 2021 [66]	Retrospective cohort	30	Cisplatin-based CRS/HIPEC. STS versus no STS.	One episode of AKI without STS, sustained at three months. No renal injury in STS group.
Zaballos, 2021 [67]	Retrospective + prospective	181	Cisplatin-HIPEC. Cilastatin/imipenem vs. no cilastatin. COI: patent holder for cilastatin use.	Fewer major Clavien–Dindo 3–4 complications, shorter ICU stay (*p* = 0.02) and hospital stay (*p* = 0.005). Lower postoperative creatinine (ANOVA *p* = 0.037). AKI by RIFLE criteria was not significantly different.
Kurreck, 2022 [68]	Retrospective (historical controlled comparison)	238	Cisplatin 75 mg/m^2^ HIPEC. STS 9 g/m^2^ bolus + 12 g/m^2^ over 6 h vs. no STS (historical control).	AKI: 6.5% (STS) vs. 30.7% (no-STS) (*p* = 0.001). STS group: prolonged ICU stay (2 vs. 1 day, *p* = 0.031) due to hypernatraemia monitoring. RRT: 0% vs. 3.1% (ns).
Krause, 2024 [69]	Retrospective	412	Predictive model for AKI risk stratification using electronic health records.	AKI incidence 8.7%. Predictive model (AUC 0.82, internal validation only) includes BMI, haemoglobin, male sex, and total intraperitoneal cisplatin dose. SMOTE applied for class imbalance. Doxorubicin excluded from final model due to collinearity with cisplatin.
Lu, 2024 [57]	Retrospective cohort	158	AKI-to-AKD transition incidence, risk factors, outcomes.	AKI 21.5% (34/158). AKD 13.3% (20/158). CKD at 90 days: 42.8% of AKD patients. 30-day mortality: 3.2% overall; 14.7% in AKI group; 14.3% in AKD group. Independent multivariable predictors of AKI: ascites (OR = 3.501), neutrophil ratio (OR = 1.195), eGFR (OR = 0.949), hypotension duration (OR = 1.030).
Gao, 2024 [56]	Retrospective cohort	480	Pseudomyxoma peritonei.	AKI: 10.6% (51/480). Hyperthermia independently associated with AKI. Full model also includes: older age (OR = 1.05), postoperative hypotension (OR = 2.09, *p* = 0.042), cisplatin-containing chemotherapy (OR = 2.75, *p* = 0.017). Intraoperative hyperthermia independent AKI risk factor.
Cheng, 2024 [70]	Retrospective	55	Cisplatin-based HIPEC.	AKI incidence: 43.6% (24/55). CKD incidence: 30.9% (17/55). Parecoxib risk factor for both AKI and CKD. Temperature > 38.5 °C independent risk factor for AKI (OR = 11.76) and CKD (OR = 6.40). One patient required temporary haemodialysis.
Gao, 2025 [71]	RCT	180	Polimixoma peritonei. Standard monitoring with urine-output-guided hydration protocol. Urine output, MAP, fluid administration.	AKI incidence within 7 days was lower in urine-guided hydration than with routine hydration (21.4% vs. 39.3%; RR 0.55; 95% CI, *p* = 0.012). Fewer major complications within 30 days in urine-guided hydration group (36.9% vs. 56.0%; RR, 0.66; 95% CI; *p* = 0.013). Adverse events did not differ between groups.
Lee, 2026 [72]	Retrospective cohort	180	Advanced ovarian cancer, interval debulking surgery with CRS/HIPEC, STS versus hydration along with acetylcysteine and mannitol.	In the STS group, 14 patients developed stage I AKI, vs. 9 in the hydration group (including 6 with stage I, 2 with stage II, and 1 with stage III). The incidence of AKI did not differ significantly between the two groups. Univariate and multivariate analyses identified estimated blood loss as a potential contributor to AKI occurrence (*p* = 0.046).

CRS, cytoreductive surgery; HIPEC, hyperthermic intraperitoneal chemotherapy; AKI, acute kidney injury; AKD, acute kidney disease; CrCl, creatinine clearance; eGFR, estimated glomerular filtration rate; KDIGO, Kidney Disease Improving Global Outcomes; RIFLE, Risk, Injury, Failure, Loss, End-stage; AKIN, Acute Kidney Injury Network; STS, sodium thiosulfate; RRT, renal replacement therapy; CKD, chronic kidney disease; ICU, intensive care unit.

## Data Availability

No new data were created or analysed in this study. Data sharing is not applicable to this article.

## Supplementary Materials

The following supporting information can be downloaded at: https://www.mdpi.com/article/10.3390/jcm15187307/s1, Table S1: Preferred Reporting Items for Systematic reviews and Meta-Analyses extension for Scoping Reviews (PRISMA-ScR) Checklist [88]; Supplementary Material S1: Electronic Search Strategy and Keywords.
