# Supplementary material for: Perioperative Fluid Management and Renal Protection in Cytoreductive Surgery with Hyperthermic Intraperitoneal Chemotherapy (CRS/HIPEC): A Scoping Review
Source: J Clin Med. 2026 Sep 20;15(18):7307. doi: 10.3390/jcm15187307 (PMC13608563; doi:10.3390/jcm15187307)
Supplement: Supplementary file 1 [file jcm-15-07307-s001.zip › Supplementary_Material_S1_Keywords.pdf]

Supplementary Material S1

Electronic Search Strategy and Keywords

The literature search was performed across electronic databases (PubMed/MEDLINE, Cochrane Central Register of Controlled Clinical Trials, EMBASE, ScienceDirect, and Scopus) covering the period from 1 January 2014 to 31 December 2025, restricted to human studies published in the English language.

1. Search Architecture and Term Combinations

The search strategy combined terms related to the surgical/chemotherapeutic procedure with terms related to hemodynamic management, fluid therapy, and renal complications/protection using Boolean operators (AND/OR).

• Procedural terms:

"cytoreductive surgery", "cytoreduction", "hyperthermic intraperitoneal chemotherapy", "HIPEC", "hyperthermic intraperitoneal chemoperfusion".

• Hemodynamic and fluid management terms:

"intraoperative management", "anaesthetic management", "anesthetic management", "haemodynamic management", "hemodynamic management", "goal-directed therapy", "goal directed fluid therapy", "GDFT", "fluid therapy", "fluid restriction", "fluid administration", "vasopressors", "norepinephrine", "cardiac output monitoring", "pulse contour analysis".

• Renal outcomes and nephroprotective terms:

"acute kidney injury", "AKI", "acute renal failure", "nephrotoxicity", "renal protection", "sodium thiosulfate", "amifostine", "cilastatin", "dexmedetomidine".

2. Database Adaptations

The core keywords and Medical Subject Headings (MeSH terms for PubMed/CENTRAL, Emtree terms for EMBASE) were adapted to match the specific indexing and search syntax of each queried database (e.g., using field tags [Title/Abstract], subject headings /exp, and Title-Abs-Key parameters) while maintaining identical thematic scope and eligibility limits.
